# Protection of Kaempferol on Oxidative Stress-Induced Retinal Pigment Epithelial Cell Damage

**DOI:** 10.1155/2018/1610751

**Published:** 2018-11-21

**Authors:** Weiwei Du, Yuanlong An, Xiangdong He, Donglei Zhang, Wei He

**Affiliations:** ^1^The School of Pharmacy, He University, Shenyang, Liaoning 110163, China; ^2^Shenyang Industrial Technology Institute of Ophthalmology, Shenyang 110163, China

## Abstract

The protection of retinal pigment epithelium (RPE) injury plays an important role in the prevention of or in delaying the pathological progress of retinal degeneration diseases, like age-related macular degeneration (AMD), diabetic retinopathy, and retinitis pigmentosa. Oxidative stress has been identified as a major inducer of RPE injury, which eventually could lead to a loss of vision. Kaempferol is a natural flavonoid widely distributed in many edible plants, fruits, and traditional medicines and has been reported to have antioxidant, anti-inflammatory, anticancer, and antimicrobial activities. The present study demonstrates that the total antioxidant capacity of kaempferol is approximately two times stronger than that of lutein which is also a natural antioxidant that is widely used in the prevention or treatment of AMD. Our data indicates that kaempferol protects human RPE cells (ARPE-19) from hydrogen peroxide- (H_2_O_2_-) induced oxidative cell damage and apoptosis through the signaling pathways involving Bax/Bcl-2 and caspase-3 molecules proofed by real-time PCR and Western blot results. Kaempferol also inhibits the upregulated vascular endothelial growth factor (VEGF) mRNA expression levels induced by H_2_O_2_ in ARPE-19 cells and affects the oxidation and antioxidant imbalanced system in ARPE-19 cells treated by H_2_O_2_ through the regulations of both the activities of reactive oxygen species (ROS) and superoxide dismutase (SOD). Furthermore, our *in vivo* experimental results show that in sodium iodate-induced retinal degeneration rat model, kaempferol could protect sodium iodate-induced pathological changes of retina tissue and retinal cells apoptosis as well as the upregulated VEGF protein expression in RPE cells. In summary, these novel findings demonstrate that kaempferol could protect oxidative stressed-human RPE cell damage through its antioxidant activity and antiapoptosis function, suggesting that kaempferol has a potential role in the prevention and therapeutic treatment of AMD or other retinal diseases mediated by oxidative stress.

## 1. Introduction

Age-related macular degeneration (AMD) is ranked as the top three ocular diseases which would lead to blindness in the world [1], and there are currently no effective treatments available for this disease. Although the exact mechanisms of AMD formation have yet to be completely understood, many studies have revealed that chronic optic injury, choroidal vascular sclerosis, and retinal pigment epithelial cell aging are closely linked to the formation of AMD [2]. More specifically, it has been found that the degeneration or dysfunction of the retinal pigment epithelium (RPE) occurs in the early pathological process in AMD and leads to the loss of vision [3]. Therefore, protection from RPE injury plays an important role in the prevention or in delaying the pathological progress of AMD.

The RPE constitutes the outer blood-retinal barrier (BRB) and is a monolayer of pigmented cells lying in the interface between the photoreceptors of the neurosensory retina and the choroidal capillary bed [4]. The RPE plays an important role not only in preventing the entrance of toxic molecules and plasma components into the retina but also in processing visual cycle and protecting against photooxidation because of its unique location and function [5]. Oxidative stress, which is a major pathological factor for cellular damage caused by reactive oxygen intermediates, has been wildly studied in AMD [6]. Reactive oxygen species (ROS) induced by oxidative stress is the main cellular reactive oxygen intermediates, which include free radicals, hydrogen peroxide, and oxygen ion from the byproducts of oxygen metabolism [7]. ROS are shown to induce cell damage and apoptosis in many tissues and cells. The previous *in vitro* studies demonstrated that oxidative stress by hydrogen peroxide (H_2_O_2_) leads to RPE cell death by causing preferential damage to its mitochondrial DNA [8]. Under normal physiological conditions, the retina demands higher oxygen supply, and therefore, high levels of cumulative irradiation surrounds the retinal, rendering RPE cells vulnerable to oxidative damage. Thus, various approaches to protecting RPE cells from oxidative stress have been investigated with the purpose of slowing down AMD progression [9].

More and more studies have shown that natural plant extracts have a certain effect on preventing and reversing AMD, especially lutein and zeaxanthin. These natural extracts have been found to help reduce and hinder the progress of AMD formation [10]. Flavonoids are antioxidants that are found abundantly in nature from a variety of plants. Among these natural flavonoids, kaempferol is a member of the flavonol subclass widely distributed in many edible plants (such as vegetables, fruits, and beans) and also in traditional herb medicines (such as chrysanthemum, *Astragalus mongholicus*, ginkgo leaf, and dry raspberry) [11]. The biological activities of kaempferol and its glycosides have been reported to have antioxidant, anti-inflammatory, anticancer, and antimicrobial effects in the tissues and cells outside the eye, as tested by the *in vitro* or *in vivo* experiments [12–17]. Kaempferol has been found to be a potent superoxide scavenger; its ability to decrease superoxide levels at low concentrations may play an important role with respect to its antioxidant activity, as the formation of superoxide anion is required for the normal production of most reactive oxygen and nitrogen species involved in oxidative stress [18].

In the present study, our team examines, for the first time, the antioxidative injury effects of kaempferol on both human and animal retinal pigment epithelial cells by *in vitro* and *in vivo* experiments in order to investigate the potential molecular mechanisms underlying such effects. Through our study, we discovered a new biological activity of kaempferol in the field of ophthalmology, suggesting that kaempferol may have the potentials for the prevention or the therapeutic function for AMD or the other retinal degeneration diseases.

## 2. Materials and Methods

### 2.1. Cells and Cell Culture

Human RPE cell line (ARPE-19) was obtained from ATCC (American Type Culture Collection). Cells were cultured in GIBCO Dulbecco's Modified Eagle Medium: supplemented with 10% fetal bovine serum at 37°C with 5% CO_2_ in a humidified atmosphere.

### 2.2. Cell Viability Assay and Oxidative Injury Model in ARPE-19 Cells

ARPE-19 cells viabilities were evaluated using 3-(4,5-dimethylthiazol-2-yl) -5-(3-carboxymethoxyphenyl)-2-(4-sulfophenyl)-2H-tetrazolium (MTS) reagent according to the manufacture's instruction (Promega, USA). Briefly, cells were plated in 96-well microplates with 1 × 10^5^ cells/well; the cells were then treated with 1 mM H_2_O_2_ for 24 h or followed by another 24 h exposure to different drugs (kaempferol, lutein, resveratrol, or anthocyanin, all of these regents are from Sigma, USA), respectively. MTS solution was added (10 *μ*L/well), and the cells were then incubated for 4 h at 37°C. The absorbance was measured at 490 nm by a microplate reader (MD Emax Plus, USA). All experiments were performed in triplicate. In each experiment, a minimum of three wells per treatment were used. The viability of ARPE-19 cells in each well was presented as percentage of the control cells (non-H_2_O_2_, normal cultured cells).

### 2.3. Total Antioxidant Capacity Assay

Ferric reducing antioxidant power (FRAP) assay is a reliable and simple method for evaluating the inhibiting ability of antioxidants in the Fenton reaction [19]. The experiment was performed according to the protocol of FRAP assay kit (Beyotime, Shanghai, China). The values of absorbance-concentrations of FeSO_4_ were used to make a standard curve; and the total antioxidant capacity was calculated as the concentration of FeSO_4_. The same concentrations of positive control Trolox (an analogue of vitamin E) or the other drugs were added in the reaction system, respectively, the reaction solution was incubated at 37°C for 3–5 minutes, and the value of absorbance was detected at 595 nm. The total antioxidant capacity of each drug was calculated according to the standard curve and compared with positive control Trolox.

### 2.4. Apoptosis Assay of ARPE-19 Cells

The antiapoptotic effect of kaempferol on ARPE-19 cells was measured by annexin V-FITC/PI double-staining assay using a flow cytometry (BD Biosciences, USA). Cells were seeded in 100 mm cell culture plates (1x10^6^ cells/plate) and were incubated overnight at 37°C with 5% CO_2_. Next day, the cells were incubated with 1 mM H_2_O_2_ for 24 h followed by 20 or 50 nM kaempferol treatment for another 24 h. After that, the cells were trypsinized and stained with annexin V-FITC and PI (from annexin V-FITC apoptosis detection kit, Nanjing Jiancheng Biology Engineering Research Institute, Nanjing, China) according to the manufacturer's protocol; the fluorescence intensity was measured by a flow cytometry (FACSCalibur, BD Biosciences, San Diego, CA, USA). The cells were divided into viable cells (annexin V^−^/PI^−^), early apoptotic cells (annexin V^+^/PI^−^), late apoptotic cells (annexin V^+^/PI^+^), and necrotic cells (annexin V^−^/PI^+^).

### 2.5. Real-Time Quantitative PCR

Total RNA was extracted from ARPE-19 cells using TRIzol Reagent (Invitrogen, USA), and the cDNA was synthesized using GoScript™ Reverse Transcription System (Promega, USA) from 2 *μ*g RNA for each sample. Real-time PCR was performed using a GoTaq® qPCR Master Mix (Promega, USA) as follows: 20 *μ*L reaction solution contained 10 *μ*L SYBR Mix, 0.4 *μ*L sense and 0.4 *μ*L antisense primers solution (from 10 *μ*M), 1 *μ*L diluted cDNA, and 8.2 *μ*L nuclease-free water. The primers used for real-time PCR were sense 5′-AGAGGTCACGGGGGCTAAT-3′ and antisense 5′-CCAGGTAACAAAACCCCACA-3′ for detecting Bcl-2 mRNA expressions, sense 5′-CAAGACCAGGGTGGTTGG-3′ and antisense 5′-CACTCC GCCACAAAGAT-3′ for detecting Bax mRNA expressions, sense 5′-TCACA GGTACAGGGATGAGGACAC-3′ and antisense 5′-TCCTGGGCAACTCAGAAG CA-3′ for detecting VEGF mRNA expressions, sense 5′-CCCATGATGTCGGA CCCTAA-3′ and antisense 5′-TGTCATGAATGAACTCGGAGGTG-3′ for detecting PEDF mRNA expressions, and sense 5′-CATGTACGTTGCTATCCAGGC-3′ and antisense 5′-CTCCTTAATGTCACGCACGAT-3′ for detecting *β*-actin mRNA expressions. The subsequent data analysis was performed using MxPro™ QPCR Software followed by comparative quantification real-time PCR. Gene expression levels were normalized to *β*-actin gene expression and compared with untreated control sample, which was assigned a value of 1 [20].

### 2.6. Western Blot Analysis

ARPE-19 cells with or without treatments were collected and lysed in a buffer containing 50 mM Tris-HCl (pH 7.5), 150 mM NaCl, 2 mM EDTA, 1% Triton, 1 mM PMSF, and protease inhibitor cocktail on ice with rotating for 30 min. Lysates were centrifuged at 12,000 rpm at 4°C for 10 min. Supernatants were saved and protein concentrations were determined by BCA protein assay kit (Beyotime, Shanghai, China). Same amount of protein lysates (60 *μ*g of each sample) were then separated with a SDS/polyacrylamide gel and transferred to a nitrocellulose membrane. The membranes were blocked with 5% nonfat dry milk at room temperature for 1 h, followed by incubation with primary antibodies (antibodies were from Santa Cruz, USA: Bax 1 : 500, Bcl-2 1 : 200, caspase-3 1 : 500, and *β*-actin 1 : 1000 were used for Western blotting) in 5% nonfat dry milk for overnight at 4°C. Membranes were washed three times with PBST and incubated with horseradish peroxidase conjugated secondary anti-mouse antibody (1 : 5000, from Pierce™, Thermo fisher scientific™, Waltham, MA, USA) at room temperature for 2 h. After additional 10 min washing with PBST, protein bands were visualized using the ECL assay kit (Beyotime, Shanghai, China). The density of each band was measured using ImageJ software. The protein expression level of each molecular was normalized to *β*-actin protein expression level and compared with untreated control, which was assigned a value of 1 [21].

### 2.7. Measurement of Intracellular Reactive Oxygen Species (ROS) Activity

ARPE-19 cells were pretreated with H_2_O_2_ (1 mM) for 24 h, followed with or without 24 h exposure to kaempferol. The activities of ROS in ARPE-19 cells were detected by reactive oxygen species assay kit (Nanjing Jiancheng Biological Research Institute, China) following manufacturer's directions. Briefly, cells were incubated with 10 *μ*M DCFH-DA reagent from the kit for 30 min in the dark at 37°C, then cells were washed twice with PBS. The intracellular ROS fluorescence intensity was determined by fluorescence microscope (Nikon ECLIPSE Ti-S, Japan).

### 2.8. Measurement of Intracellular Superoxide Dismutase (SOD) Activity

The activities of SOD in ARPE-19 cells were detected using a superoxide dismutase assay kit (Nanjing Jiancheng Biological Research Institute, China). Briefly, ARPE-19 cells were pretreated with 1 mM H_2_O_2_ for 24 h, followed by a 24 h exposure to kaempferol. The cells were repeatedly frozen and thawed several times, making sure the cells were thoroughly broken. The activities of SOD in ARPE-19 cells were detected to assess the antioxidative capacities (unit/per mg protein) using the superoxide dismutase assay kit following the manufacturer's directions [22].

### 2.9. Animal Experiments

#### 2.9.1. Sodium Iodate-Induced Retinal Degeneration Rat Model

In this study, adult Sprague-Dawley rats, weighing 200 g to 250 g, were obtained from the Laboratory Animal Resource Center of the Liaoning Province. Animals were fed and treated in standard condition; experimental protocol in this study was approved by the Animal Experimentation Ethics Committee of He University in China.

Sodium iodate-induced retinal degeneration animal model has been widely used for studying retinal degeneration diseases and drug treatment effects [23]. In our study, five rats were used for each experimental group. The experimental animals were divided into four groups: normal control group, solvent group, sodium iodate model group, and kaempferol pretreatment group. Sodium iodate (from Sigma-Aldrich, St. Louis, MO) was dissolved in sterile normal saline at a stock concentration of 4% (*w*/*v*). The rats were anesthetized by intraperitoneal (i.p.) injection of chloral hydrate. 50 *μ*L 0.3% kaempferol was injected by intravitreal injection at 3 days before sodium iodate injection. Sodium iodate (40 mg/kg) was given via the sublingual vein. The control group was the rats injected with the same volume of normal saline. After 7 days of normal diet water, the eyeballs were taken for follow-up experiments.

#### 2.9.2. Retinal Hematoxylin-Eosin Staining

Hematoxylin-eosin (HE) staining was performed according to the conventional methods [24]. Briefly, eyeballs were fixed in 4% paraformaldehyde, after 1 h along the limbal corneal cut out crystal, and were transferred to the gradient sucrose solution (from 5%, to 10%, to 20% sucrose solution, for 2 h in each solution at room temperature) then were kept in 30% sucrose solution at 4°C overnight. Next day, the tissues were frozen in liquid nitrogen and were stored in −20°C refrigerator. The sections were stained with hematoxylin and eosin dyes, and the images were taken by a light microscope (Nikon, ECLIPSE Ni, Japan).

#### 2.9.3. Stretched Preparation of RPE-Choroid-Sclera Complex Tissue

The freshly removed eyeballs were fixed in 4% polyformic acid for 0.5 h, then the cornea was opened along the corneal edge, and the eyeball was fixed in 4% polyformic acid for another 2 h, then was transferred in PBS for 0.5 h. Removing the connective tissue around the eyeball under the microscope, the anterior segment was cut off along the edge of the sclera and made 6–8 radial incisions by cutting to the optic nerve root. The retinal neurosensory layer was removed to confirm that RPE-choroidal-sclera complex could be fully covered, glycerine seal, it will be observed by laser confocal microscope (Zeiss, Jena, Germany).

#### 2.9.4. Retinal TUNEL Staining

The apoptosis of retinal cells was examined using a terminal deoxynucleotidyl transferase (TdT) dUTP nick-end labeling (TUNEL) IHC kit (Roche, 11684817910, USA) according to the manufacturer's protocol. At room temperature, after incubation with 0.3% Triton X-100 for 20 minutes for permeabilization, the sections were incubated with TdT reaction buffer for 10 minutes, then the sections were incubated with TdT reaction cocktail for 60 minutes at 37°C and washed twice with 3% BSA in PBS for 2 minutes each time. The sections were visualized, and the images were captured on an inverted fluorescent microscope (Nikon, ECLIPSE Ni, Japan) [25].

#### 2.9.5. Immunofluorescence Staining

The eyeballs were enucleated and immersed in 4% paraformaldehyde for 60 minutes. After fixation, the eye cups were dehydrated using a gradient sucrose solution and embedded in OCT compound (Sakura Finetek, USA). Cryosections (8 *μ*m thickness) were obtained along the temporal-nasal axis through the optic nerve head. The tissue specimens were blocked and permeabilized simultaneously with 3% BSA in 0.3% Triton X-100 for 1 hour at room temperature and then incubated with primary antibodies specific for VEGF (1 : 1000, Abcom, UK) and RPE65 (1 : 500, Abcom, UK) at 4°C overnight, followed by incubation with FITC-labeled secondary antibodies (Abcam) for 1 hour at room temperature. Cell nuclei were counterstained with 49-6-diamidino-2-phenylindole (DAPI). The retinal sections were examined, and the images were captured using an inverted fluorescence microscope (Nikon ECLIPSE Ni, Japan) [26].

### 2.10. Statistical Analysis

All experiments were performed at least three times. Values in the experiments described were presented as mean ± standard deviation (SD). Means were tested for statistical significance using Statistical Product and Service Solutions (SPSS) software, and *p* < 0.05 was considered as statistical significance.

## 3. Results

### 3.1. Kaempferol Decreases H_2_O_2_-Induced Cytotoxicity in ARPE-19 Cells

**Figure 1:**
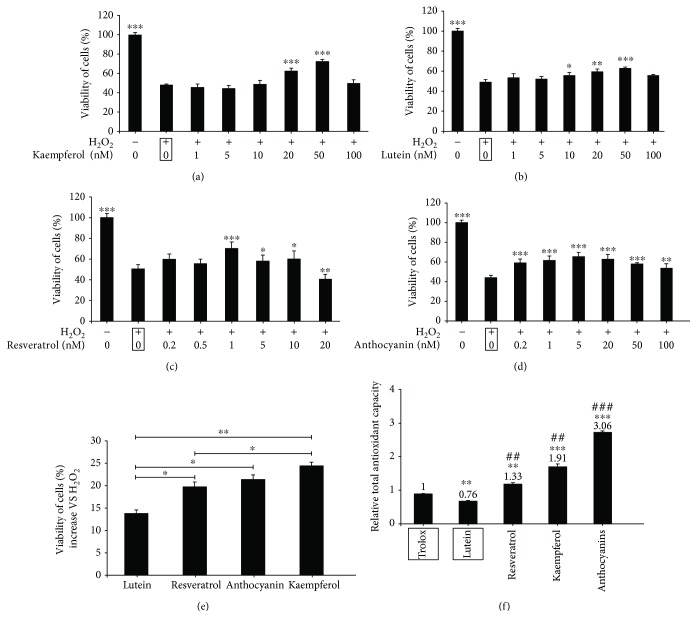
Kaempferol increased the cell viabilities in H_2_O_2_-treated ARPE-19 cells. (a)–(e) The viability of cells was measured by MTS assay; the viability of cells was expressed as a percentage of that of the control group (nontreated cells). (a) ARPE-19 cells were treated with 1 mM H_2_O_2_ for 24 h. The cells were exposed to the individual concentrations of kaempferol; (b)–(d) ARPE-19 cells were treated with 1 mM H_2_O_2_ for 24 h; the cells were then exposed to the individual concentrations of lutein (b), resveratrol (c), or anthocyanin (d) for 24 h without H_2_O_2_. (e) Comparison of the protective effects of lutein, resveratrol, kaempferol, and anthocyanin by MTS assay. (f) Comparison of total antioxidant capacity of kaempferol, lutein, anthocyanin, and resveratrol (#*p* < 0.05, ##*p* < 0.01, ###*p* < 0.001 vs. the lutein sample). All the data are shown as mean ± SD, ^∗^*p* < 0.05, ^∗∗^*p* < 0.01, ^∗∗∗^*p* < 0.001 vs. the samples which are marked by black frames.

To investigate if kaempferol has the protective effects on oxidative stressed-RPE cells *in vitro*, we first confirmed that kaempferol at concentrations 1–100 nM had no apparent effects on ARRE-19 cell viabilities (data not shown) measured by MTS assay; we then examined the cell viabilities of H_2_O_2_-induced ARPE-19 cells followed with 1–100 nM kaempferol treatments. In Figure 1(a), the results showed that ARPE-19 cells were treated with 1 mM H_2_O_2_ for 24 h, the cell viability decreased to about 48% compared with that of nontreated control cells, and after treatment with H_2_O_2_, at the concentration of 50 nM, kaempferol revealed the maximum cytoprotective effect; it significantly increased the cell viability from 48% to 72%. We also found that pretreatment with kaempferol had the similar protective effects on H_2_O_2_-induced cytotoxicity in ARPE-19 cells (data not shown).

It is well known that lutein, resveratrol, and anthocyanin are all natural antioxidants, and it has been reported that these natural products can prevent cell damages and organ aging [27, 28]; these natural antioxidants have also shown to have preventative and therapeutical effects on RPE cell damages [29]. To compare the cytoprotective ability of kaempferol to these naturel products on ARRE-19 cells, the same experiment as above was performed by treatment the cells with different concentrations of lutein, resveratrol, and anthocyanin instead of kaempferol. In Figures 1(b)-1(d), the results demonstrated that the concentrations of lutein, resveratrol, and anthocyanin, which induced the maximum protective effects on H_2_O_2_-induced cytotoxicity, were 50 nM, 1 nM, and 5 nM, respectively. In Figure 1(e), lutein (50 nM), resveratrol (1 nM), anthocyanin (5 nM), and kaempferol (50 nM) were compared with their abilities of cytoprotective effect on ARRE-19 cells; the cell viabilities increased about 13.8 ± 2.69%, 19.7 ± 1.31%, 21.4 ± 4.03%, and 24 ± 3.85%, respectively, compared with H_2_O_2_-treated samples; kaempferol and anthocyanin showed the strongest protecting effect on ARPE-19 cells against oxidative stress-induced cell damages. Furthermore, we measured the total antioxidant capacity of kaempferol and the other three natural antioxidants; in Figure 1(f), the results showed that the order of total antioxidant capacity was anthocyanin > kaempferol > resveratrol > lutein. These data suggested that although the total antioxidant capacity of kaempferol is about 2/3 of anthocyanin, it has a similar protective effect on H_2_O_2_-induced cytotoxicity in ARRE-19 cells.

### 3.2. Kaempferol Inhibits H_2_O_2_-Induced ARPE-19 Cell Apoptosis

**Figure 2:**
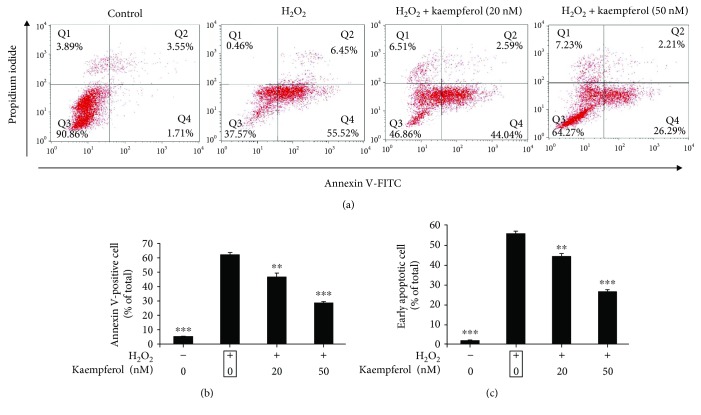
Antiapoptotic effects of kaempferol on ARPE-19 cells against oxidative stress induced by H_2_O_2_. (a) ARPE-19 cells with or without H_2_O_2_ treatment for 24 h, then cells were treated with or without kaempferol (20 or 50 nM/mL) for another 24 h, the cells were then stained with annexin V/PI double staining and analyzed by flow cytometry. The percentage of cells in each quadrant is presented. (b) The quantification of annexin V-positive cells (Q2 + Q4) was calculated for each group cells and are shown in the bar graph. (c) The quantification of early apoptotic cells (Q4) was calculated for each group cells and are shown in the bar graph. The data in (b) and (c) are shown as mean ± SD, ^∗^*p* < 0.05, ^∗∗^*p* < 0.01, ^∗∗∗^*p* < 0.001 vs. the samples treated with H_2_O_2_ alone (marked by black frame).

To investigate the protective effect of kaempferol against H_2_O_2_-induced cell apoptosis, ARPE-19 cells were incubated with 1 mM H_2_O_2_ for 24 h and then cells were exposed to kaempferol for 24 h without H_2_O_2_ existence. The percentages of apoptotic cells were determined with flow cytometry assay using annexin V-FITC/PI double staining method, and the results were shown in Figure 2. The distribution of population of annexin V-FITC/PI double positive staining cells is shown in Figure 2(a); the percentage of apoptotic cells (annexin V positive cells) or early apoptosis cells (annexin V positive/PI negative cells) was calculated and were presented in Figures 2(b) and 2(c), respectively. With the treatment of 1 mM H_2_O_2_, the percentage of apoptotic cells increased to 61.97 ± 1.68% (untreated control cells: 5.26 ± 0.25%), and treatment with 20 or 50 nM kaempferol, the percentages of apoptotic cells (annexin V positive cells) decreased to 46.63 ± 2.74% and 28.5 ± 1.09%, respectively (Figure 2(b)). With the treatment of kaempferol, the percentages of early apoptotic cells (annexin V positive/PI negative cells) decreased from 55.52 ± 1.28% (H_2_O_2_ only treatment cells) to 44.04 ± 1.49% (20 nM) and 26.29 ± 1.67% (50 nM), respectively; untreated control cells was 1.71 ± 0.37% (Figure 2(c)). These data show that kaempferol could mainly protect early apoptotic cells in H_2_O_2_-induced ARPE-19 cells.

### 3.3. The Antiapoptotic Effects of Kaempferol on Oxidative Stressed RPE Cells through the Bcl-2/Bax/Caspase-3 Signaling Pathway

**Figure 3:**
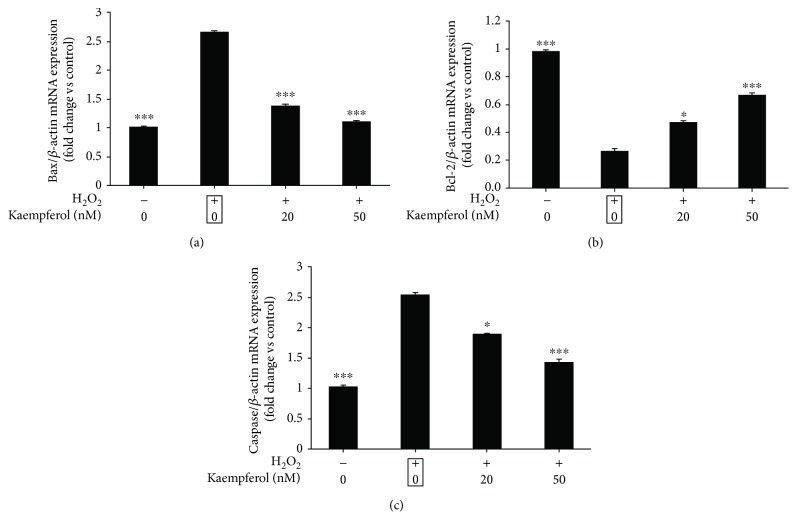
Effects of kaempferol on mRNA expression levels of Bax, Bcl-2, and caspase 3 in H_2_O_2_-treated ARPE-19 cells. ARPE-19 cells were pretreated with or without H_2_O_2_ (1 mM) for 24 h, followed by a 24 h exposure to kaempferol (20 or 50 nM). The mRNA expression levels of Bax (a), Bcl-2 (b), and caspase-3 (c) for each sample were measured by real-time PCR, and the fold changes of each gene are shown in the bar graph compared with the control sample (nontreated cells, value as 1). The data are shown as mean ± SD, ^∗^*p* < 0.05, ^∗∗^*p* < 0.01, ^∗∗∗^*p* < 0.001 vs. the sample marked with black frame (H_2_O_2_ treatment alone).

Oxidative stress could induce the cell apoptosis mainly through FasL signaling or mitochondrial signaling; some reports have revealed that H_2_O_2_-induced ARPE-19 cells apoptosis is related to the mitochondrial apoptotic signaling which is involving the proapoptotic protein Bax, the antiapoptotic protein Bcl-2, and the downstream protein caspase-3 [30]. We then measured the mRNA and protein expression levels of Bax/Bcl-2/caspase-3 in H_2_O_2_-pretreated ARPE-19 cells followed by kaempferol treatment. In Figure 3, the ARPE-19 cells were pretreated with 1 mM H_2_O_2_, then the cells were treated with or without kaempferol for 24 h, the mRNA levels of Bax, Bcl-2, and caspase-3 were measured by real-time PCR, and the fold change of gene expression levels compared with nontreated control cells was shown in Figure 3(a) (Bax), 3B (Bcl-2), and 3C (caspase-3), respectively. The results demonstrate that H_2_O_2_ treatment increased both of the Bax and caspase-3 mRNA expression levels up to 2.6-fold (*p* < 0.05) but decreased the Bcl-2 mRNA expression up to 0.27-fold. Treatment with 20 or 50 nM kaempferol significantly decreased the upregulated Bax and caspase-3 mRNA expression levels induced by H_2_O_2_, meantime, significantly increased the downregulated Bcl-2 mRNA expression levels induced by H_2_O_2_.

**Figure 4:**
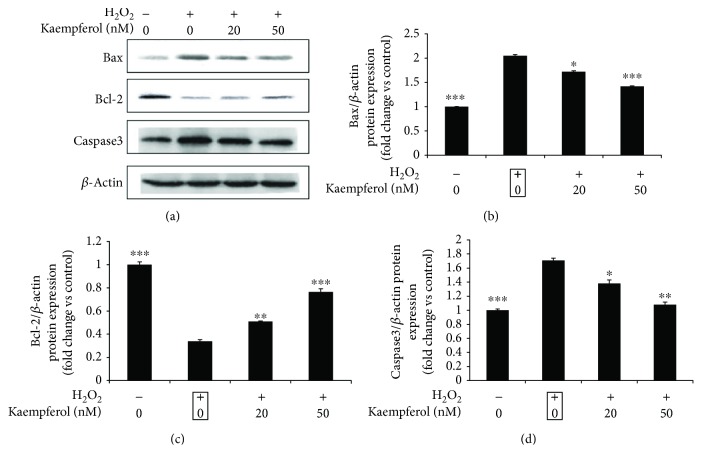
Effects of kaempferol on the protein expression levels of Bcl-2, Bax, and caspase 3 in H_2_O_2_-treated ARPE-19 cells. ARPE-19 cells were pretreated with or without 1 mM H_2_O_2_ for 24 h, followed by a 24 h exposure to kaempferol (20 or 50 nM). (a) The cell lysates of control (non-H_2_O_2_ treatment), H_2_O_2_-treated, and kaempferol-treated samples were analyzed by Western blot assay with the antibodies of Bcl-2, Bax, caspase 3, and *β*-actin, respectively. Quantification data of these protein expression levels were measured and normalized to *β*-actin expression levels. The fold changes vs. control sample were presented in bar graph from (b)–(d), respectively. Data are shown as mean ± SD, ^∗^*p* < 0.05, ^∗∗^*p* < 0.01, ^∗∗∗^*p* < 0.001 vs. the sample marked with black frame (H_2_O_2_ treatment alone), *n* = 3.

To test if these altered levels of mRNA could also be detected at the protein levels, similar experiments as above were performed, and the protein expression levels of Bax, Bcl-2, and caspasae-3 were measured by Western blot (Figure 4(a)); the fold changes of these protein expressions were calculated and presented in Figures 4(b)–4(d). The Western blot results show that kaempferol decreased the upregulated Bax and caspase-3 protein expression levels induced by H_2_O_2_, and kaempferol increased the downregulated Bcl-2 protein expression levels induced by H_2_O_2_ responding to the same results as that of mRNA experiments. These results indicated that kaempferol protects the ARPE-19 cells apoptosis induced by oxidative stress through the mitochondrial signaling.

### 3.4. Kaempferol Affects H_2_O_2_-Induced Intracellular ROS and SOD Activities in ARPE-19 Cells

**Figure 5:**
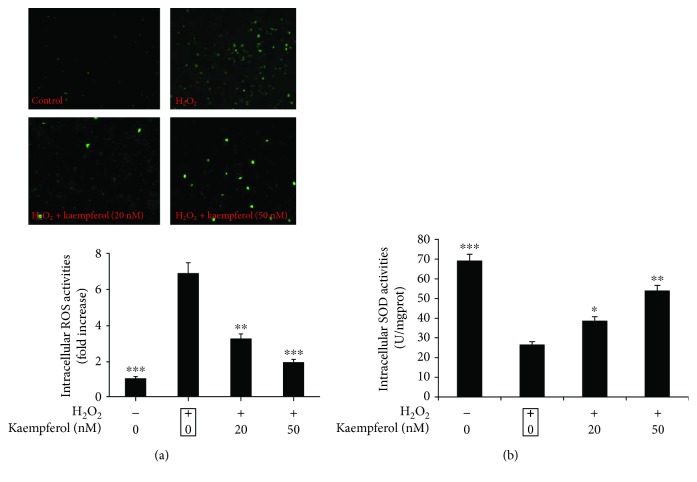
Kaempferol significantly decreased the ROS activity while increased SOD activity in H_2_O_2_-treated ARPE-19 cells. (a) ARPE-19 cells were pretreated with or without 1 mM H_2_O_2_ for 24 h, followed by a 24 h exposure to kaempferol. The intracellular reactive oxygen species (ROS) activities were measured by fluorescence microscope. (b) ARPE-19 cells were pretreated with or without 1 mM H_2_O_2_ for 24 h, followed by a 24 h exposure to kaempferol; the activities of cellular SOD were measured by a SOD activity assay kit. The SOD levels were normalized against per milligram protein. Data were shown as mean ± SD (*n* = 3). ^∗^*p* < 0.05, ^∗∗^*p* < 0.01, ^∗∗∗^*p* < 0.001 vs. H_2_O_2_ only treated group (marked by black frame).

Many studies have demonstrated that oxidative stress leads to reactive oxygen species (ROS) production beyond the limits of clearance in vivo and causes the oxidation and antioxidant system imbalance, resulting in functional and morphological impairments in retinal pigment epithelium (RPE), endothelial cells, and retinal ganglion cells [31]. Meanwhile, superoxide dismutase (SOD) is an important antioxidant enzyme in cells, it can remove oxygen-free radicals and protect cells from oxidative injury. Superoxide dismutase (SOD) activity level can reflect the cellular antioxidant ability. Therefore, we were interested to investigate if kaempferol could affect the activities of ROS and SOD in ARPE-19 cells during the H_2_O_2_-induced oxidative stress [32]. In Figure 5, the ROS and SOD activities were measured in ARPE-19 cells after H_2_O_2_ only or followed by kaempferol treatment. In Figure 5(a), the data showed that H_2_O_2_ induced an increase of intracellular ROS activities about 7-fold compared with non-H_2_O_2_-treated sample, and treatment with 20 or 50 nM kaempferol significantly decreased the upregulated ROS activities induced by H_2_O_2_ up to the normal levels. Meantime, H_2_O_2_ treatment decreased the intracellular SOD activities about 38% compared with non-H_2_O_2_-treated sample, and treatment with 20 or 50 nM kaempferol partly increased the downregulated SOD activities induced by H_2_O_2_ (Figure 5(b)). Thus, the above results illustrate that kaempferol could affect the oxidation and antioxidant imbalanced system induced by H_2_O_2_ through the regulations of both activities of ROS and SOD in ARPE-19 cells.

### 3.5. Kaempferol Protects Pathological Changes of Retina Tissue in Sodium Iodate-Induced Retinal Degeneration Rats

Sodium iodate (NaIO_3_, as a kind of stable oxidant, has been shown to effectively induce retinal degeneration; this model is widely used because of its reproducibility and controllable retinal damage degree [33]. The main characteristic of the retinal degeneration induced by sodium iodate is the regional loss of retinal pigment epithelium; it can occur the morphological features of similar pattern atrophy [34]. It is considered that sodium iodate focuses on the retinal pigment epithelial cells in the retina, leading to the necrosis of the retinal pigment epithelium and then the choroid capillary atrophy and the whole retinal degeneration [35].

**Figure 6:**
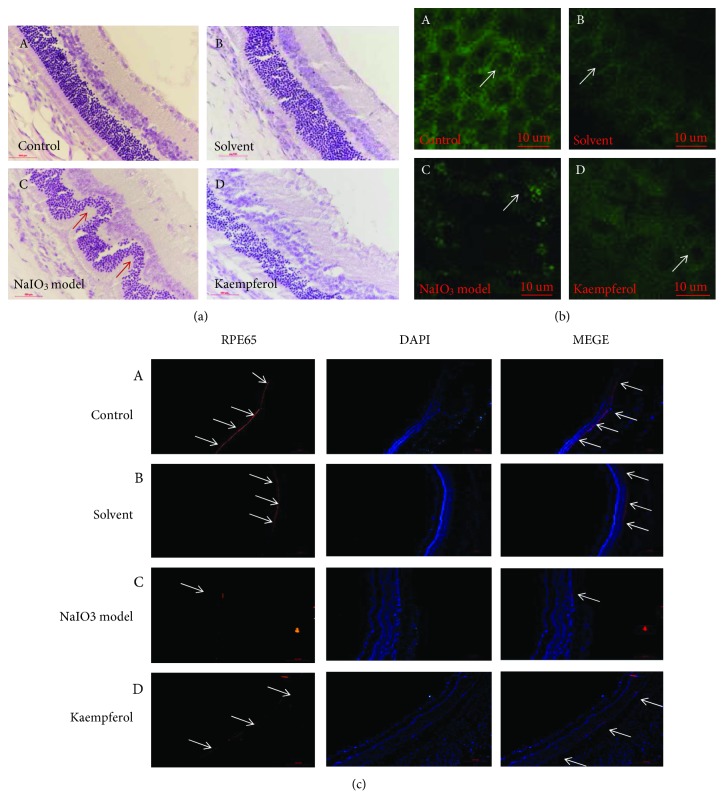
Pretreatment of kaempferol significantly improved sodium iodate-induced retinal damage in SD rats. The rats were anesthetized by intraperitoneal injection of chloral hydrate, sodium iodate (40 mg/kg) was given via the sublingual vein, and the kaempferol was injected into vitreous cavity 3 days before sodium iodate injection. After 7 days of sodium iodate injection, each group rats were analyzed by (a) retinal HE staining; (b) histological analysis of RPE cell layer using confocal microscope through stretched preparation of RPE-choroid-sclera complex tissue; (c) retinal immunofluorescence staining with RPE65 antibody. Experimental groups were: (A) normal control; (B) solvent control; (C) NaIO_3_ injection model; (D) kaempferol pretreatment before NaIO_3_ injection. Scale bar is 500 *μ* m.

To observe whether the kaempferol has a protective effect on retinal degeneration once it gets to the retinal tissue *in vivo*, SD rats were anesthetized by intraperitoneal (i.p.) injection of chloral hydrate; sodium iodate (40 mg/kg) was given via the sublingual vein; and the kaempferol was injected into vitreous cavity before sodium iodate injection. After 7 days of sodium iodate injection, HE staining, TUNEL staining, RPE65 staining, and VEGF-immunofluorescence staining were performed on frozen sections of retinal tissues. In Figure 6(a), HE staining results showed that the retinal structure of blank solvents treated group had no significant changes compared with the control group (nontreated group), and the retinal tissue of the model group (treated with sodium iodate) was severely damaged; the outer nuclear layer cells were disorganized and thinned, and a wavy change of the retina could be visibly observed. Compared with the model group, kaempferol pretreated group's outer nuclear layer mildly disordered, the outer nuclear layer thickness reduction was not obvious, and the wave shape change had been greatly improved. The disorganized retinal tissue in sodium iodate-treated group and the protective effect of kaempferol on disorganized retinal tissue were also observed by observing RPE cell layers under a confocal microscope (Figures 6(b), A–6(b), D); the RPE cells of control group and blank solvents group were no obvious changes and arranged regularly (Figures 6(b), A and 6(b), B). In Figure 6(b), C, the model group results showed the RPE cell apoptosis, fusion, and intercellular structure disorder. Compared with the model group, kaempferol drug group nuclei appeared part of apoptosis; cells arranged relatively neat (Figure 6(b), D).

RPE65 protein is the specific expression protein of retinal pigment epithelial cells, it is closely related to the susceptibility of retinal photo injury. The immunofluorescence results showed that the RPE65 protein of retinal pigment epithelial cells in the control group and blank solvents group was expressed in the whole RPE cell layer (Figures 6(c), A and 6(c), B). Compared with the normal group, Figure 6(c), C results showed that the expression level of the specific protein RPE65 in the RPE cells of the NaIO3-treated model group was significantly reduced, showing that NaIO3 caused serious damage of RPE cells. The RPE65 protein expression of RPE cells in the kaempferol group got an obvious improvement compared with that of the NaIO3-treated model group (Figure 6(c), D); these results suggested that the kaempferol can protect the RPE cells damage induced in sodium iodate-induced retinal degeneration rats.

### 3.6. Kaempferol Inhibits NaIO3-Induced Retinal Cell Apoptosis *In Vivo*

**Figure 7:**
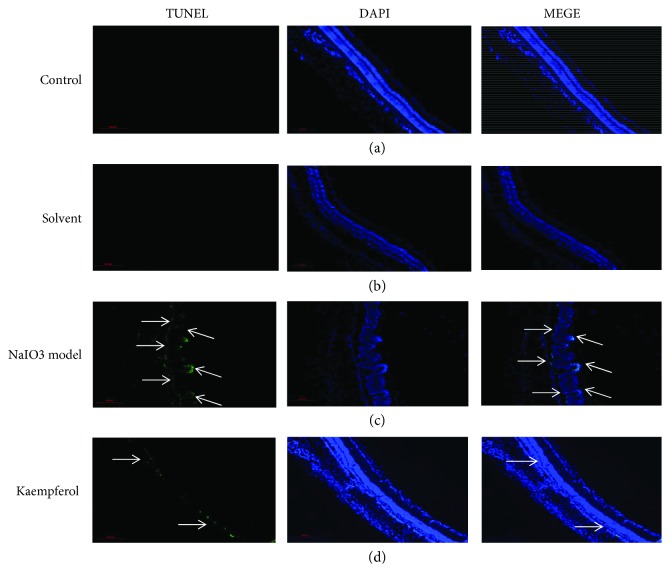
Kaempferol attenuated the apoptosis of retinal cells in sodium iodate injected SD rats. The frozen sections were used for TUNEL staining to detect the apoptosis of retinal cells, and the nucleus was stained by DAPI. Experimental groups were (a) normal control; (b) solvent control; (c) NaIO_3_ injection model; (d) kaempferol pretreatment before NaIO_3_ injection. Scale bar is 500 *μ*m.

The antiapoptosis activity of kaempferol on ARPE-19 cells had been observed by *in vitro* experiments (Figures 2 and 3), so it was interesting to investigate if kaempferol has the protective effect on NaIO3-induced retinal cells apoptosis *in vivo*. Using immunofluorescence staining, TUNEL-labeled apoptosis positive cells were analyzed in the control group, NaIO3-induced group, kaempferol pretreated group, or solvent pretreated group rats, and DAPI staining were used for nucleus labeling. In Figure 7, the results showed that almost no apoptotic cells were found in the control group and blank solvents group (Figures 7(a) and 7(b)); after 7 days of treatment by NaIO3, a large number of apoptotic cells were observed in retinal pigment epithelium cells and outer nuclear layer cells (Figure 7(c)). In contrast, apoptotic cells were significantly improved in kaempferol pretreated group (Figure 7(d)). This is consistent with the previous reports that the main targets of the toxicity of sodium iodate are on RPE cells and outer nuclear layer cells.

### 3.7. The Effects of Kaempferol on VEGF and PEDF Expression

**Figure 8:**
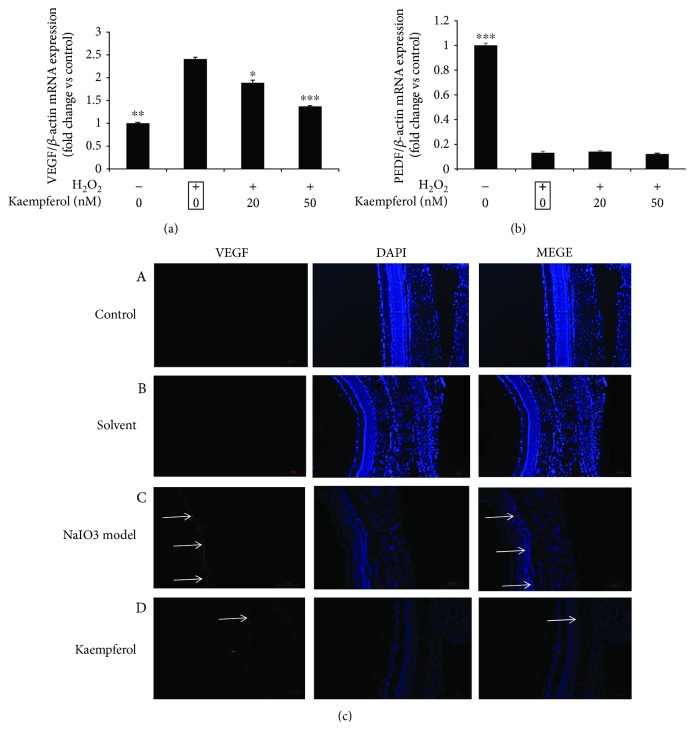
Effect of kaempferol on the expressions of VEGF and PEDF. (a) ARPE-19 cells were pretreated with or without 1 mM H_2_O_2_ for 24 h, followed by a 24 h exposure to kaempferol (20 or 50 nM). The mRNA expression levels of VEGF and PEDF were analyzed by real-time PCR, and the fold changes of each gene were shown in the bar graph compared with the control samples (nontreated cells, value as 1). The data are shown as mean ± SD, ^∗^*p* < 0.05, ^∗∗∗^*p* < 0.001 vs. the sample marked with black frame (H_2_O_2_ treatment alone). (b) Effect of kaempferol on the expressions of VEGF in retinal tissue. Retinal immunofluorescence staining with VEGF antibody was performed in each experimental groups: (A) normal control; (B) solvent control; (C) NaIO_3_ injection model; (D) kaempferol pretreatment before NaIO_3_ injection. Scale bar is 500 *μ*m.

Some groups have reported that oxidative stress in RPE cells could induce the upexpression of vascular endothelial growth factor (VEGF), which is an effective angiogenic growth factor and plays an important role in the formation of wet AMD [36]. On the other hand, pigment epithelium-derived factor (PEDF), which was first found in RPE cells, has been reported having the functions of antioxidative damage, anti-inflammation, and neuroprotection [37]. More specifically, the imbalance of the ratio of VEGF to PEDF is though closely related to the angiogenesis [38]. First, we investigated if kaempferol has any effects on VEGF and PEDF mRNA expressions in H_2_O_2_-treated ARPE-19 cells. In Figure 8(a), the results showed that in H_2_O_2_-treated ARPE-19 cells, VEGF mRNA expression was significantly increased about 2.4-fold compared with that of untreated control cells, while treatment with kaempferol significantly decreased the upregulated expression levels of VEGF mRNA induced by H_2_O_2_; the VEGF mRNA expression levels of kaempferol-treated cells were about 1.89-fold (20 nM kaempferol) and 1.37-fold (50 nM kaempferol), respectively, compared with untreated control cells. In H_2_O_2_-treated ARPE-19 cells, PEDF mRNA expression level was significantly decreased about 87% compared with untreated control cells, but treatment with kaempferol had no effects on the downregulated PEDF expression levels induced by H_2_O_2_ (Figure 8(b)).

Next, we investigated if kaempferol could protect the upregulated VEGF expressions induced by oxidative stress *in vivo*. In sodium iodate-induced retinal degeneration rats, 7 days after NaIO3 injection, the upregulated VEGF protein expressions were observed mainly on RPE cells compared with that of untreated control group (Figure 8(c)), and pretreatment with kaempferol decreased the upregulated VEGF protein expressions in sodium iodate-induced retinal degeneration rats.

## 4. Discussion

In recent years, the flavonoids from natural plants have become a subject of interest due to their potent antioxidant capacity and low toxicity, which, in return, makes them highly effective as potential therapeutic agents. According to their molecular structures, flavonoids are divided into six classes: flavones, flavanones, flavonols, isoflavones, anthocyanidins, and flavanols [39]. Derived from edible plants, the flavonoids mainly contain anthocyanins and flavonols. Some studies have shown that the anthocyanins from blueberry and other fruits could improve the oxidative stress-induced retinal damages [40]. However, anthocyanins are highly unstable and are very susceptible to degradation. Flavonols, from edible plants, mainly include quercetin, kaempferol, myricetin, and morin. Among these flavonols, quercetin is the most common flavonol, which has been reported to have pharmacological functions on the ocular tissues; but so far, there have not been any reports published with respect to the functions of kaempferol in the context of retinal pigment epithelium. Although, the extracts from certain traditional Chinese medicines (TCMs: such as dry raspberry, chrysanthemum, semen cuscutae, *Astragalus mongholicus*, and ginkgo leaf), which contain kaempferol, have been demonstrated as having the function of improving vision and are used in clinical therapeutic treatments (Chinese pharmacopoeia, 2015 version). The exact mechanisms underlying these effects have not been fully elucidated. In the present study, we first discovered that kaempferol has the protective effects on oxidative stress-induced RPE cells tested by *in vitro* and *in vivo* experiments, but it has no protective effects on the other ocular tissue cells (like corneal epithelial cells or lens epithelial cells, data are not shown). These data suggested that kaempferol can protect the retina from oxidative damage together with other flavonoids found in natural plants, and the possible mechanisms may be through its protective effects on RPE cells.

As previously mentioned, oxidative stress plays an important role in the pathogenesis of AMD. Thus, it would be of great significance to explore the oxidative damage of RPE cells in order to examine the pathogenesis and treatment of AMD. Recent studies have confirmed that kaempferol has antioxidant properties [41], and we compared the total antioxidative capacities with kaempferol to some other nature antioxidants (including lutein, resveratrol, and anthocyanin), which have been reported as having the protective function on RPE cells [42–44]. We found that kaempferol and anthocyanin had similar antioxidative capacity, which was stronger than that of lutein and resveratrol (see Figure 1(e)). Meanwhile, kaempferol and anthocyanin also showed stronger antioxidative damage abilities than that of lutein and resveratrol on H_2_O_2_-induced ARPE-19 cells (see Figure 1(f)). Furthermore, kaempferol had shown no effects on ARPE-19 cells proliferation in our experiments (data not shown).

In order to confirm if kaempferol has the antiapoptotic effects on H_2_O_2_-induced ARPE-19 cells, annexin V and PI double staining were performed, and the percentage of early apoptotic cells (annexin V positive, while PI negative cells) and total apoptotic cells (annexin V positive cells) were analyzed by FACS assay. The results showed that kaempferol had antiapoptotic effects mainly on the early apoptotic cells (Figure 2(b)). Many studies have illustrated that the apoptosis of RPE cells induced by oxidative stress are highly associated with the mitochondria-related apoptotic signaling, which typically involves the increasing of pro-apoptotic proteins (like Bax and caspase-3) and decreasing the antiapoptotic proteins expression (like Bcl-2 ect) [45]. More specifically, the ratio of Bax/Bcl-2 plays a role in the production of apoptosis in cells. In our study, we found that the treatment of ARPE-19 cells by kaempferol decreased the Bax and caspase-3 protein expressions and increased Bcl-2 protein expression level, and the real-time PCR results that revealed the alters of these protein expressions were due to the alters of their mRNA transcriptional levels of these molecules regulated by kaempferol treatments. The detailed mechanisms of which signaling pathways are involved in these regulations are still undergoing further studies in our lab.

Sodium iodate is a known toxin to selectively induce RPE damage by oxidative stress and consequently induce retinal degeneration [34]. We have investigated the effects of kaempferol on retinal lesions in sodium iodate-induced retinal degeneration in rats. In Figures 6–8, the results showed that after 7 days intravenous injection of sodium iodate, an extensive disruption was observed in the outer nuclear layer (ONL), photopigment layer (PPL), and retinal pigment epithelium (RPE). In contrast, pretreatment with kaempferol ameliorated sodium iodate-induced retinal degeneration in rats by counteracting oxidative stress through decreasing the proportion of folded retina, decreasing the apoptosis of RPE cells and ONL cells and the upregulated VEGF protein expression in RPE cells. These *in vivo* experimental results suggested that, once kaempferol could get into the retinal tissue, it may protect the retinal oxidative stress through those functions mentioned in this study.

Vascular endothelial growth factor (VEGF) is widely distributed in human and animal brain, liver, kidney, eye, and other tissues. Under normal circumstances, there is a low level of VEGF expression in the retinal cells (including RPE cells and retinal pigment endothelial cells). Under regular physiological conditions, the expression of low concentration of VEGF in the eyes is necessary to maintain the integrity of the ocular blood vessels. However, overexpression of VEGF in ocular tissue will cause the production of ocular neovascularization and is associated tightly to the wet AMD or DR [46]. Xu et al. recently reported that kaempferol inhibited VEGF expression and in vitro angiogenesis of human retinal endothelial cells under diabetic-like environment [47]. Our experiments demonstrated that kaempferol could inhibit the upregulated VEGF mRNA expression levels, which are induced by oxidative stress in ARPE-19 cells, and could also reduce the upregulated VEGF protein expression in RPE cells observed by *in vivo* experiments in sodium iodate-induced retinal degeneration in rats (Figure 8). These results suggested that kaempferol potentially has the function of preventing and treating the angiogenesis of AMD or DR.

In summary, our *in vitro* and *in vivo* experimental results showed that kaempferol could protect retinal cells (especially RPE cells) from oxidative stress damage. The protective function of kaempferol on oxidative stressed-RPE cells is expressed through its antiapoptotic effect and the downregulation of VEGF expression function. As a result, kaempferol may be able to serve as a potent therapeutic agent for the treatment of retinal degeneration diseases by intravitreal injection. Whether an oral route given dose of kaempferol has a similar retinal protective effect remains to be further studied.

## Data Availability

The data used to support the findings of this study are available from the corresponding author upon request.
